# Adipose derived pericytes rescue fractures from a failure of healing – non-union

**DOI:** 10.1038/srep22779

**Published:** 2016-03-21

**Authors:** T. Tawonsawatruk, C. C. West, I. R. Murray, C. Soo, B. Péault, A. H. R. W. Simpson

**Affiliations:** 1 The Department of Orthopaedic Surgery, The University of Edinburgh, UK; 2 BHF Centre for Vascular Regeneration, Scottish Centre for Regenerative Medicine, The University of Edinburgh, 5 Little France Drive, Edinburgh, EH16 4UU, UK; 3 The Department of Plastic Surgery, NHS Lothian, Edinburgh, UK; 4 The Department of Orthopaedic Surgery, Ramathibodi Hospital, Mahidol University, Bangkok, Thailand; 5 Orthopaedic Hospital Research Centre, David Geffen School of Medicine, University of California at Los Angeles, California, USA; 6 Division of Plastic and Reconstructive Surgery, University of California, Los Angeles, CA 90095. 7Orthopaedic Hospital Research Center, University of California, Los Angeles, CA 90095.

## Abstract

Atrophic non-union is attributed to biological failure of the fracture repair process. It occurs in up to 10% of fractures, results in significant morbidity to patients, and treatment often requires complex reconstructive procedures. We tested the ability of human bone derived marrow mesenchymal stem cells (MSC), and human adipose derived pericytes (the native ancestor of the MSC) delivered percutaneously to the fracture gap to prevent the formation of atrophic non-union in a rat model. At eight weeks, 80% of animals in the cell treatment groups showed evidence of bone healing compared to only 14% of those in the control group. Radiographic parameters showed significant improvement over the eight-week period in the cell treatment groups, and histology confirmed bone bridges at the fracture gap in the both treatment groups. The quality of bone produced and its biomechanical properties were significantly enhanced in both treatment groups. The results from this study demonstrate that MSC and pericytes have significant bone regeneration potential in an atrophic non-union model. These cells may have a role in the prevention of atrophic non-union and could enable a paradigm shift in the treatment of fractures at high risk of failing to heal and developing non-union.

The majority of fractures heal without any complications, however a significant proportion fail to heal resulting in delayed union and subsequent non-union. The frequency of non-union is between 5–10% and the incidence is 19 per 100,000^1^. Non-union is associated with patient factors such as smoking^2^, diabetes and obesity, and non-patient factors such as high energy injury^3^. Non-union causes profound additional morbidity for patients, who describe their lives as being ‘on hold’ for the years that their bone remains un-united. There is a great need therefore to identify and treat patients destined to develop non-union at an early stage after fracture. Further, established non-unions pose clinicians significant challenges and many current treatments require complex reconstructive procedures. Therefore, minimally invasive and effective treatments to address this condition at an early stage would be extremely beneficial.

Regenerative Medicine is defined as “the process of replacing or regenerating human cells, tissues or organs to restore or establish normal function”. Currently under investigation are many novel therapeutic treatments that use autologous and allogeneic cells to treat a range of pathologies including bone loss. Mesenchymal Stem Cells (MSC) represent ideal candidates for addressing bone loss and regeneration due to their ability to differentiate into osteoblasts. However, MSC also have many other functions that make them suitable therapeutic candidates including secretion of growth factors, stimulation of angiogenesis and modulation of the immune system^4^,^5^. Most studies that have focused on MSC have used bone marrow as the primary source as this was the tissue from which they were originally described^6^,^7^,^8^. However the use of bone marrow MSC may be limited due to the morbidity associated with harvest and the small numbers of cells available by this method. Furthermore, the inherent heterogeneity of these populations makes their study difficult. In contrast, adipose tissue is an abundant and readily accessible source of stem cells with minimal morbidity using techniques such as liposuction^9^,^10^,^11^. The amount of tissue that can be retrieved is vast in comparison to bone marrow, and the frequency of stem cells within adipose tissue has been shown to be 500 times greater than that of bone marrow per unit of mass^12^. This has allowed a better understanding to be developed of the subpopulations of cells within the stromal vascular fraction (SVF). The relative abundance of these cells has also enabled these subpopulations to be purified to homogeneity, which has facilitated a better understanding of their individual function and therapeutic potential^4^,^13^,^14^.

It is now accepted that microvascular pericytes represent the *in-vivo* precursors of the MSC^13^,^15^,^16^,^17^,^18^,^19^. Using fluorescence activated cell sorting (FACS) it has been demonstrated that prospectively purified pericytes demonstrate mesenchymal potential that is equal, and in many cases superior to conventional MSC^4^ (Fig. 1). In addition to any potential functional advantage, there are also regulatory advantages that using a defined and purified population of cells – such as pericytes - may provide in their clinical translation. The clinical use of stem cells has prompted stringent guidance from the US Food and Drug Administration (FDA) and European Regulation in 2011 for the preparation and processing of cells in accordance with current good manufacturing guidelines. Approved stem cell technology must be evaluated with regards to safety, purity, identity, potency and efficacy prior to biologic licensing and clinical use, all of which are facilitated by using a defined and pure population of cells^20^.

The aim of this study was to examine the ability of human adipose derived pericytes to heal a fracture destined to progress to an atrophic non-union in a rat tibia model, and to compare this to conventional human bone marrow MSC. Bone marrow MSC were chosen due to their recognized ability to repair and regenerate bone. Furthermore, their ability to treat established non-union has been described^21^.

## Results

### Reproducible induction of non-union in a rat tibial model

Our previously validated method of inducing atrophic non-union in which rats demonstrate no evidence of radiological union 8 weeks following tibial osteotomy was applied to a group of 17 rats^22^,^23^. There was no evidence of healing at the fracture gap as confirmed by radiography at 3 weeks. At this point 5 were treated with MSC injection, 5 were treated with pericyte injection, and 7 were given PBS (control). There were no adverse reactions following the injections.

### Percutaneous administration of MSC and pericytes protected rats from the development of non-union

X-ray images were taken weekly to evaluate the degree of healing at the fracture site (Fig. 2A). A significantly greater radio-opacity and significantly greater increase in callus area were noted in the pericyte and MSC groups at weeks 4, 6 and 8 compared to the PBS controls (p < 0.05). A significantly higher proximal callus index was observed in both the MSC and pericyte groups compared to the PBS controls, 8 weeks following injection (p < 0.05). There were no differences between any of the groups at the earlier time points (weeks 0 and 2), and no differences were detected between MSC and pericyte at any time point, in any of the parameters measured (Fig. 2B).

2 independent, blinded experts (orthopaedic surgeons with experience of interpreting experimental murine x-rays) reviewed the x-ray images taken 8 weeks following injection to determine whether there was evidence of persistent non-union or union (Fig. 2C). In the groups treated with MSC (n = 5), 4 had radiographic evidence of union versus 1 with persistent non-union (P-value < 0.05, Fishers’ exact test compared to control). In the pericyte group (n = 5), 3 had union versus 1 with delayed union (confirmed histologically) and 1 with non-union, and in the PBS group (n = 7), 1 had union versus 6 with non-union (P > 0.05).

### Percutaneous administration of MSC and pericytes enhanced bone quality at the fracture gap

MicroCT demonstrated that there was no difference in trabecular thickness (Tb.Th) between any of the groups. Bone Volume Density (BV.TV) was significantly greater in the MSC v PBS (p < 0.05). Bone Mineral Density (BMD) was significantly higher in both MSC and pericyte groups compared to the PBS controls (P < 0.05). There were no significant differences between MSC and pericyte groups in any of the parameters measured (P > 0.05) (Fig. 3A).

### Histological assessment of the fracture gap demonstrated improved mature bone formation following percutaneous administration of MSC and pericytes

Both MSC and pericytes demonstrated significantly greater amounts of mature bone tissue at the fracture gap when compared to the PBS controls (p < 0.05). There were no differences in the area of cartilage tissue, fibrous tissue or undifferentiated tissue between any of the groups studied (P > 0.05) (Fig. 3B).

### Percutaneous administration of MSC and pericytes resulted in stable bony constructs

There were no differences between MSC and pericyte treated groups for any of the tested parameters tested including ultimate load, ultimate stress, Young’s modulus and toughness (P > 0.05) (Fig. 3C). The tibias from the PBS injection group were found to be macroscopically unstable and not suitable for biomechanical testing.

### Cell tracing studies indicated a regenerative process independent of direct engraftment of transplanted cells

Histological sections from the fracture gap were examined for the presence of MSC and pericytes by staining with Cm-Dil and Anti-human nuclear antibody. After 8 weeks there were no human cells found in the fracture gaps in any of the animals examined (Fig. 3D).

## Discussion

The therapeutic effects of percutaneous cell injections were assessed in a fracture model destined to develop atrophic non-union. 7 out of 10 animals in the cell injection groups had evidence of union and 1 had evidence of delayed union at eight weeks after injection, while in the control group, only one of seven animals had bone union at eight weeks (the one animal in the control group that united was thought to have done so because the periosteum had not been fully removed posteriorly). The bone radiopacity indicated that the degree of bone mineralisation and percentage increase of callus area were significantly improved following pericyte injection. Semi-quantitative analysis of the histomorphology of the fracture gap showed that there was significantly more bone tissue in the pericyte injection group compared to the control group. Similarly the parameters from micro-CT analysis showed significant increases: the BMD was significantly higher in the pericyte treatment group. The mechanical properties of bone following MSC and pericyte injection were comparable. These results suggested that the percutaneous injection of MSC and pericytes at the fracture gap 3 weeks after fracture improved fracture healing in an atrophic non-union model.

**Figure 1:**
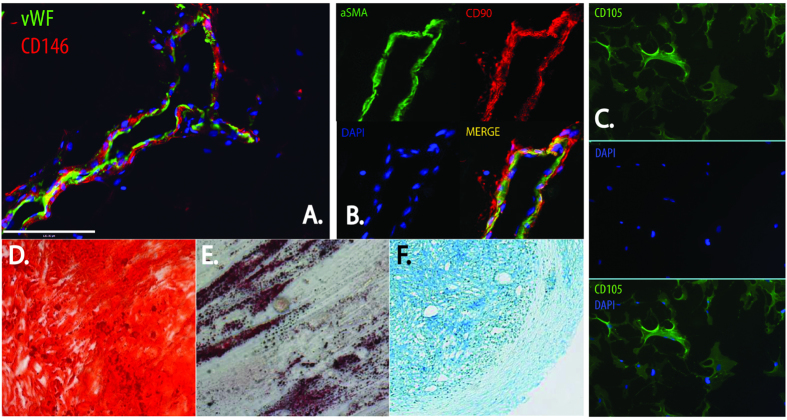
Pericytes at the origin of MSC. (**A**) Pericytes expressing CD146 reside on the abluminal surface of endothelial cells expressing vWF. (**B**) αSMA expressing pericytes co-express MSC markers including CD90. (**C**) *In-vitro* pericytes express MSC markers including CD105. Cultured pericytes are multipotent as seen by their ability to differentiate into bone (**D)** (Alizarin red staining ×10), fat (**E)** (Oil Red O staining × 10), cartilage (**F)** (Alcian blue staining × 5).

**Figure 2:**
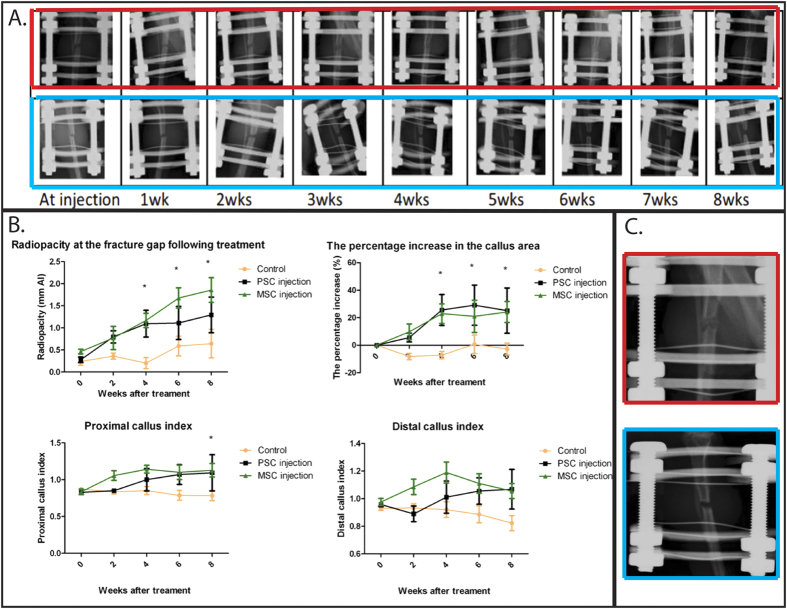
Assessment of fracture healing using serial radiography. (**A**) Serial radiographs demonstrating the development of bony non union (top row) and the progression to atrophic non-union in our model, (**B**) Quantitative assessment of callus in the two experimental groups (pericytes and MSC injection) and the control group, (**C**) Radiographic evidence of union (top) versus persistent non-union (bottom) at 8 weeks.

**Figure 3:**
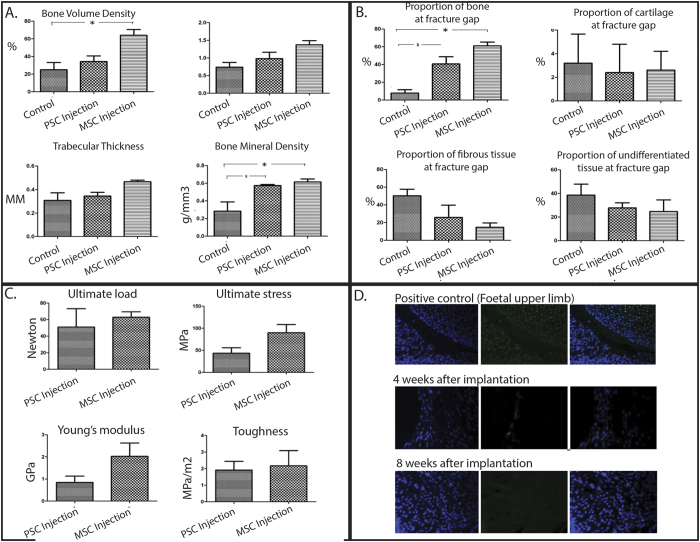
Multi-parametric analysis of fracture healing. (**A**) Quantitative Micro-CT analysis, (**B**) Histological assessment of the components present at the fracture gap, (**C**) Mechanical testing using 4 point bending, (**D**) Cell tracing reveals no transplanted cells in the fracture gap at 8 weeks.

Although it has been reported that pericytes are capable of *in vivo* mineralization following intramuscular implantation^13^ and bone regeneration in a critical size calvarial defect model^24^, the current study is the first to demonstrate the preventative benefit of pericytes in a clinically relevant model of fractures destined to develop atrophic non-union. These findings support the use of pericytes for bone regeneration. It has been proposed that pericytes are perivascular ancestors of human MSC^15^, and the *in vivo* localisation of these cells has been demonstrated^13^. *In vitro*, they demonstrate all the characteristics of conventional MSC including proliferation and multi-lineage differentiation^13^ (Fig. 1). It has been reported that pericytes can be isolated from several tissues and can be purified using cell surface markers including positive expression of CD146, NG2, PDGFRb and negative expression of CD45, CD56, CD31 and CD34^15^,^17^,^19^. In this study adipose tissue was used as a source because it is readily available and easy to harvest and could be used as an alternative source of MSC. Notably, sufficient pericytes for clinical application could be obtained without culture expansion but purely by cell selection^24^.

Pericytes can be harvested from adipose tissue in sufficient numbers for immediate autologous use without the requirement for culture. It is therefore possible to use pericytes for cell based therapies within an intraoperative approach because sufficient numbers of cells can be sorted immediately from adipose tissue and implanted back to the fracture site. Although the stromal vascular fraction of human adipose tissue (SVF) from lipoaspirate can be used intraoperatively to generate autologous cell based therapies for bone repair^25^, it has been shown that the potential for bone formation of SVF was inferior to sorted pericytes from matched patients in a model of intramuscular implantation in SCID mice^26^. Pericytes demonstrated significant increased expression of bone specific markers when quantified using immunohistochemistry, and micro-CT assessment of the implanted structures demonstrated significantly greater bone volume and bone mineral density in the pericyte group compared to SVF^26^. In addition, as pericytes are a homogeneous and defined cell population, they are likely to be more consistent in their function compared to SVF. SVF contains a heterogeneous mixture of haematopoietic cells, stem/progenitor cells, fibroblasts, smooth muscle cells and endothelial cells, with significant variation in the relative proportion of each of these cell types between patients^4^,^24^. This variation in composition is likely to result in differences in function, something that may be minimised by the use of a homogenous cell source such as pericytes.

The specific underlying molecular mechanisms of bone regeneration in the cell treatment groups have not been evaluated in this study. However, there are at least two possible mechanisms; 1) as precursor cells or 2) through trophic effects^15^. Pericytes can undergo multi-lineage differentiation similar to MSC and may improve bone regeneration via direct differentiation into osteoblast cell lineages^13^. Pericytes also secrete several growth factors known to contribute to bone healing and vasculogenesis such as heparin binding epidermal growth factor (HB-EGF), basic fibroblast growth factor (bFGF), platelet derived growth factor-B chain (PDGF-BB), vascular endothelial growth factor (VEGF), keratinocyte growth factor (KGF), angiopoietin 2 (ANG2) and thrombopoietin (TPO)^15^,^27^. The use of immunocompetent animals and xenografted cells in this study makes engraftment unlikely. As the injected cells were not present at week eight post-injection, this suggested that the broad mechanism of action in this model was one of paracrine action and trophic support rather than direct contribution of the cells as progenitors. This is most encouraging and suggests that pericyte-mediated prevention of non-union will be even more efficient in an autologous or even in an allogeneic setting, the two scenarios most likely to be faced in the clinic.

Pericytes isolated from adipose tissue have advantages over conventional bone marrow derived MSC as they are a defined and homogenous population, which can be isolated from the fat tissue abundantly without the necessity for culture expansion. Pericytes may be considered as an alternative to bone marrow derived MSC for cell therapies to promote fracture healing in fractures at risk of progressing to atrophic non-union.

## Conclusion

Cellular therapy delivering MSC or pericytes via percutaneous injection enhanced bone healing in bones destined to develop atrophic non-union. Adipose derived pericytes are a promising candidate cell type that had equal performance compared to bone marrow MSC in promoting recovery and regeneration in rescuing fractures from progressing to atrophic non-union.

## Materials and Methods

### Experimental design

The main goal of this study was to determine the potential of 2 distinct mesenchymal stem cell populations in preventing a fracture progressing to non-union in a previously validated rat model. All rats underwent identical procedures and were then allocated to either control (n = 7) or one of 2 treatments groups (each with n = 5). Serial plain radiography, micro-CT and immunohistochemistry was performed on all groups and interpreted by 2 independent experts who were blinded to treatment allocation.

### Isolation and culture of cells

#### Adipose derived pericytes

Human adipose tissue was collected from healthy female adult donors (n = 6) undergoing cosmetic liposuction procedures with prior written consent. Ethical approval for the collection of tissue and subsequent research was granted by the South East Scotland Research Ethics Committee 3 (SESREC03), reference number 10/S1103/45. Collection of tissue was performed in accordance with the approved guidelines and with informed consent from all subjects.

**Figure 4:**
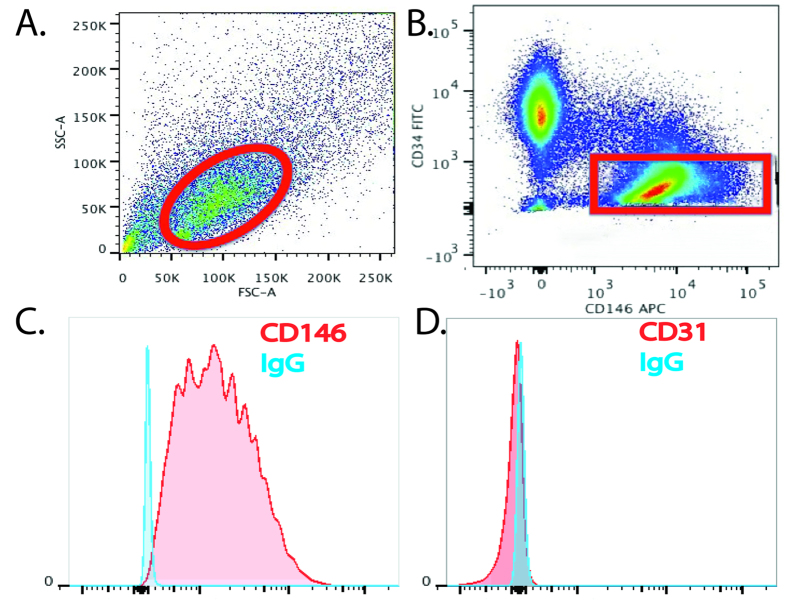
Purification and analysis of pericytes by flow cytometry. (**A**) Pericytes can be purified from the SVF of adipose tissue by FACS. Cells (circled in red) are selected on an initial FSC v SSC dot plot. (**B**) Following removal of dead cells, haematopoietic (CD45+) and endothelial cells (CD31+), pericytes (red box) can be selected based on their unique phenotype (CD146+, CD34−, CD31−, CD45−). (**C,D)** Pericytes maintain a stable phenotype over extended periods of culture (CD146+, CD31−).

Pericytes were isolated from adipose tissue by Flow Activated Cell Sorting (FACS) using a FACS Aria II (BD Biosciences) based on our established protocols^4^. Briefly, adipose tissue was enzymatically digested with type II collagenase (1 mg/ml, Sigma-Aldrich) for 30 mins in a shaking waterbath at 37 °C to obtain the Stromal Vascular Fraction (SVF). SVF was then stained with the following antibodies; CD146-Alexa647 (1:100, AbD Serotec, Raleigh, NC), CD45 APC-cy7, CD31-FITC, and CD34-PE (1:100, all from BD Biosciences, San Jose, CA). Pericytes were sorted to homogeneity based on the following phenotype CD146+, CD45−, CD34−, CD31− (Fig. 4A,B).

Immediately following FACS, pericytes were seeded onto 0.1% gelatin coated wells at a density of 20,000 cells/cm2 in EGM-2 media (Lonza) in a humidified incubator with 20% 0_2_, 5% CO_2_ at 37 °C. When confluent, cells were detached from the cultureware using 0.25% trypsin and split at a ratio of 1:6 and cultured in DMEM + 20%FCS + 1%Pen/Strep for all subsequent passages. Media was changed 3 times per week. Purity of pericyte cultures was confimed by flow cytometry (Fig. 4C,D).

#### Bone marrow MSC

hMSCs were isolated from the femoral heads of patients undergoing hip replacement operations. These tissues were obtained under informed consent and in accordance with the approval given by the local research ethics committee (LREC 2002/1/22). The established protocol for isolating hMSC in this study has been previously reported^6^. Bone marrow and cancellous bone from the femoral head were removed and digested using a collagenase solution (Collagenase type II, Sigma-Aldrich, UK). Nucleated cells were seeded in a 75 cm^2^ flask incubator with 20% 0_2_, 5% CO_2_ at 37 °C for 48 hours. After incubation, the culture medium containing non-adherent cells was removed and the remaining adherent cells were washed with 1xPBS three times before addition of fresh basal medium. Primary isolated adherent cells were maintained with addition of fresh culture medium (DMEM + 20%FCS + 1%Pen/Strep) every three days until the cells reached 80% confluence. All experiments were performed using MSC and pericytes at passage 4–6.

### Animal model and intervention

All procedures and protocols were conducted following approval by South East Scotland Research Ethics Committee and the UK Home Office, and in accordance with the animal (Scientific Procedure) Act 1986. Seventeen Wistar rats underwent the procedure to induce an atrophic non-union. The method of inducing atrophic non-union has been reported previously^22^,^23^. The tibia was exposed and stabilized with an external fixator. A mid shaft osteotomy was created with a 1 mm gap between the ends of the exposed bone. To induce atrophic non-union, the periosteum was stripped for a length of one-diameter of the tibia both proximal and distal to the osteotomy site.

After the operation, animals were randomly allocated either to the pericyte treatment group (n = 5), the bone marrow derived MSC treatment group (n = 5) or to the control group (n = 7). 5 × 10^6^ cells were percutaneously injected into the fracture gap 3 weeks after operation. The cell suspension was injected slowly to avoid leakage. After injection, animals were monitored to ensure ongoing health and welfare with particular attention paid to weight, behaviour, and condition of the injected site.

### Outcomes

The new bone formed in the osteotomy gap was analysed using a range of outcome measures: Serial radiography was performed to record changes in radiopacity at the fracture gap and the size of the callus. The outcome measures were evaluated 8 weeks after injections. These measures were;

1. Radiographic assessment of union versus non-union by 2 independent blinded experts. The callus index was derived by dividing the total width of the bone including any new callus by the original width of the bone.
2. Quantitative Micro-CT analysis to determine Bone Mineral Density (BMD), Bone Volume Density (BV/TV) and Trabecular thickness (Tb.Th).
3. Histological assessment to determine the relative amount of bone, cartilage, fibrous and undifferentiated tissue at the fracture gap.
4. Mechanical testing using four-point bending to determine the ultimate load, ultimate stress, Young’s modulus and toughness.
5. Cell tracing studies using Cm-Dil and Anti-human nuclear antibody to determine the contribution of transplanted cells to the repair and remodelling.

### Statistical analysis

The difference in numbers of bone unions between the cell injection groups and the control group was determined using Fishers’ exact test. The mean differences in fracture progression parameters including radiopacity, proximal and distal callus index, the percentage increase of callus area were tested using repeated ANOVA and the post-hoc analysis was performed using Bonferroni multiple comparisons test. The measurements from histology and micro-CT were also tested using one way ANOVA. Post-hoc analysis was performed using Bonferroni multiple comparisons test. The biomechanical parameters were tested using unpaired t-tests. A p-value of < 0.05 was considered statistically significant.

## Additional Information

**How to cite this article**: Tawonsawatruk, T. *et al.* Adipose derived pericytes rescue fractures from a failure of healing – non-union. *Sci. Rep.* **6**, 22779; doi: 10.1038/srep22779 (2016).
